# Effect modification of age and hypertension on cancer and prevalence of self‐reported stroke – A cross‐sectional study

**DOI:** 10.1002/cam4.5964

**Published:** 2023-04-21

**Authors:** Ronda Lun, Joseph R. Shaw, Danielle Carole Roy, Deborah Siegal, Tim Ramsay, Yue Chen, Dar Dowlatshahi

**Affiliations:** ^1^ Division of Neurology, Department of Medicine The Ottawa Hospital Research Institute Ottawa Ontario Canada; ^2^ University of Ottawa School of Epidemiology Ottawa Ontario Canada; ^3^ Division of Hematology, Department of Medicine The Ottawa Hospital Ottawa Ontario Canada

**Keywords:** cancer, cerebrovascular disease, effect modification, prevalence, stroke

## Abstract

The objective of this study was to examine the effect modification of age on the relationship between cancer and prevalence of self‐reported stroke. We used cross‐sectional data from the 2015–2016 iteration of the Canadian Community Health Survey. A multivariable logistic regression model was used to assess the association between cancer and self‐reported stroke. Covariates were assessed for effect modification using the maximum likelihood estimation method. We analyzed 86,809 subjects; the prevalence of self‐reported stroke was 1.11%. The odds ratio for the association between cancer and self‐reported stroke was 1.26 (95% CI 0.98–1.61) after adjusting for age, sex, dyslipidemia, hypertension, diabetes, heart disease, education, and household income. Age and hypertension were found to be effect modifiers, and the association between cancer and self‐reported stroke was stronger in younger adults and in those without hypertension. These results suggest that cancer‐associated strokes may have unique underlying mechanisms compared to conventional strokes.

## Abstract 2

Age has been found to be an effect modifier of the relationship between cancer and stroke. The association between cancer and stroke is strongest in young individuals (<40 years) and without a history of hypertension.

## 1 INTRODUCTION

Stroke is a leading cause of adult disability.^1^, ^2^ The prevalence of stroke in Canada was estimated to be approximately 405,000 in 2013, representing 1.15% of the population.^3^ This is projected to increase to 726,000 by 2038, due to the aging population, a rise in cardiovascular risk factors, and increased survival from recent advances in stroke treatment.^3^, ^4^, ^5^

The presence of cancer is an understudied risk factor for stroke.^6^ Patients with cancer have a higher risk for stroke compared to the general population. This risk persists for decades after cancer diagnosis.^7^, ^8^ The association between cancer and stroke may vary by age.^9^ Younger patients are more likely to experience nonconventional stroke mechanisms, and classification as cryptogenic etiology may prompt investigations searching for malignancy.^10^ Previous studies have reported a higher than expected prevalence of cancer among young patients with stroke compared to the general population with similar age.^11^ However, in older populations, due to the increased prevalence of cardiovascular risk factors, the causal relationship between cancer and stroke is less clear. Assessments of potential interactions between cancer and age on the prevalence of stroke in the general population have not been previously performed.

Our objective is to compare the prevalence of self‐reported stroke among patients with and without cancer and to examine the effect of age on this relationship.

## 2 METHODS

### 2.1 Data source

This study used cross‐sectional data from the 2015–2016 iteration of the Canadian Community Health Survey (CCHS), conducted by Statistics Canada.^12^ This dataset is publicly accessible; no approval was necessary from regional institutional research boards.

The target population of the CCHS was Canadians aged 12 years or more in all provinces and territories, but excluded those living on First Nations Reserves, members of the Canadian Forces, institutional residents, children living in foster care, and residents of remote regions, which represented approximately 2% of the target population.^13^ We included only adult subjects ≥18 years. Our exposure of interest was a composite of current or prior diagnosis of cancer. CCHS survey questions that were used to ascertain a prior diagnosis of cancer included “Do you have cancer?” and “Have you ever been diagnosed with cancer?” The outcome of interest was a respondent's self‐report of “suffering from the effects of stroke”, which was referred to as “prevalence of self‐reported stroke”. This was ascertained by asking the question “Do you suffer from the effects of a stroke?” in the survey. The CCHS survey questionnaire did not collect information on the timing of self‐reported stroke relative to the reported prior diagnosis of cancer.” Any subjects with missing information with respect to cancer/stroke status or covariates were excluded from analysis.

### 2.2 Variables of interest

Covariates of interest identified from the literature included sex, smoking status, dyslipidemia, hypertension, heart disease, diabetes, racial background, level of education, and annual household income. We recategorized age into seven subgroups: 18–29, 30–39, 40–49, 50–59, 60–69, 70–79, and ≥80. We recategorized smoking status into current smoker, former smoker, or nonsmoker. Based on the median income of $61,400 in Canada, we dichotomized household income into <$60,000 annually or ≥$60,000.^14^ We also dichotomized education level into those who obtained postsecondary education or those who did not, and cultural/ racial background as White or non‐White.

### 2.3 Statistical analysis

The prevalence of self‐reported stroke was calculated for those with and without cancer and according to covariates. Logistic regression analysis was performed to evaluate the association between having cancer and the prevalence of self‐reported stroke. Effect modification was assessed using likelihood ratio test: effect modification was explored by examining interaction terms between cancer and the individual covariates. An interaction term was considered to be significant if the *p*‐value for the interaction term was <0.05 and assessment for final inclusion in the model was based on between‐model comparisons using the likelihood ratio test—only models that offer significantly better (*p* < 0.05) goodness of fit than the original model were selected. Confounding was defined as a change of greater than 10% in the parameter estimate for the association of interest. Crude and adjusted odds ratios (ORs) with 95% confidence intervals (CIs) were presented. Stratified logistic regression analysis was further conducted according to detected effect modifiers (age and hypertension).

The CCHS uses a complex survey design with features including stratification, clustering, unequal selection probability, and multistage sampling. To account for this, we calculated adjusted weights (relative weights divided by the square root of the average design effect of the CCHS) for included participants, which account for both sampling weights and average design effect when calculating point estimates and variance estimates, using methods previously described by Chen et al.^15^

All statistical analyses were performed using SAS version 9.4.

## 3 RESULTS

We analyzed 86,809 survey respondents (Table S1). There were 1287/86,809 (1.11%) subjects that answered “yes” to the question “Do you suffer from the effects of a stroke”. The prevalence of self‐reported stroke was higher in those who reported having cancer compared to those without cancer (2.88% vs. 1.00%, respectively; *p* < 0.0001). The prevalence of self‐reported stroke increased with age; male sex, former smoking status, dyslipidemia, hypertension, diabetes, heart disease, lower education level, white racial background, and lower household income were also significantly associated with an increased prevalence of self‐reported stroke (Table S1).

The crude OR for the association between cancer and self‐reported stroke was 2.93 (95% CI 2.33–3.69, *p* < 0.0001). The overall association between cancer and self‐reported stroke was no longer statistically significant (OR 1.26, 95% CI 0.98–1.61, *p* = 0.062) after adjusting for age, sex, dyslipidemia, hypertension, diabetes, heart disease, education, and household income (Table 1).

**TABLE 1:** Crude and adjusted weighted multivariable logistic regression models for the prevalence of self‐reported stroke in relation to active or history of cancer in Canadians. Results are presented as odds ratios (ORs) with 95% confidence intervals (95% CI).

Predictors	Crude		Adjusted^a^	
	ORs (95% CI)	*p*‐Value	ORs (95% CI)	*p*‐Value
Cancer				
Yes	2.93 (2.33–3.69)	<0.0001	1.26 (0.98–1.61)	0.062
No (reference)	–	–	–	–
Age (years)				
18–29 (reference)	–	–	–	–
30–39	0.53 (0.29–0.97)	0.038	0.54 (0.30–0.99)	0.046
40–49	1.90 (1.23–2.93)	0.0036	1.60 (1.03–2.48)	0.036
50–59	2.19 (1.44–3.32)	0.0002	1.33 (0.87–2.04)	0.19
60–69	5.60 (3.83–8.19)	<0.0001	2.31 (1.55–3.47)	<0.0001
70–79	10.81 (8.39–15.80)	<0.0001	3.25 (2.15–4.91)	<0.0001
≥80	18.43 (12.44–27.32)	<0.0001	4.77 (3.10–7.35)	<0.0001
Sex				
Male	1.25 (1.06–1.48)	0.008	1.30 (1.09–1.55)	0.0029
Female (reference)	–	–	–	–
Dyslipidemia				
Yes	4.13 (3.48–4.91)	<0.0001	1.27 (1.04–1.54)	0.018
No (reference)	–	–	–	–
Diabetes				
Yes	5.31 (4.41–6.40)	<0.0001	1.74 (1.42–2.13)	<0.0001
No (reference)	–	–	–	–
Heart disease				
Yes	10.75 (8.97–12.88)	<0.0001	3.51 (2.87–4.30)	<0.0001
No (reference)	–	–	–	–
Education level				
No postsecondary education	2.13 (1.81–2.52)	<0.0001	1.32 (1.11–1.57)	0.0021
Postsecondary education (reference)	–	–	–	–
Annual household income				
<$60,000	2.52 (2.13–2.98)	<0.0001	1.44 (1.20–1.72)	0.0001
≥$60,000 (reference)	–	–	–	–

^a^ Adjusted for cancer, age, sex, dyslipidemia, hypertension, diabetes, heart disease, education, and household income.

All covariates were tested for effect modification using the likelihood ratio test; only hypertension and age were found to be significant effect modifiers of the relationship between cancer and stroke. Age and hypertension significantly modified the relationship between cancer and prevalence of self‐reported stroke (*p*‐values for the interaction terms of cancer with age and hypertension were both <0.0001). When stratified by age and hypertension, we found that the association between stroke and cancer tended to be greater among younger age groups, especially for adults under the age of 40 years, and was consistently attenuated by the presence of hypertension (Table 2). We did not identify a statistically significant association between cancer and self‐reported stroke among respondents with hypertension and age ≥40 years. Among respondents without hypertension, a statistically significant association between cancer and self‐reported stroke was found only for respondents that were 50–59 years of age (*p* = 0.023).

**TABLE 2:** Association between cancer and prevalence of “suffering from the effects of a stroke” according to effect modifiers: age and hypertension. Odds ratios (ORs) with 95% confidence intervals (95%CI) reflect the odds of self‐reported stroke in patients with an active or prior diagnosis of cancer.

	Adjusted ORs^a^ (95% CI)	*p*‐Value
Age group 1: 18–29		
No hypertension	49.98 (18.21–137.2)	<0.0001
Hypertension	29.51 (9.63–90.45)	<0.0001
Age group 2: 30–39		
No hypertension	38.48 (13.28–111.50)	<0.0001
Hypertension	22.72 (7.18–71.87)	<0.0001
Age group 3: 40–49		
No hypertension	1.17 (0.26–5.23)	0.84
Hypertension	0.69 (0.15–3.25)	0.64
Age group 4: 50–59		
No hypertension	2.52 (1.13–5.59)	0.023
Hypertension	1.49 (0.65–3.39)	0.35
Age group 5: 60–69		
No hypertension	1.68 (0.95–2.99)	0.076
Hypertension	0.99 (0.58–1.72)	0.98
Age group 6: 70–79		
No hypertension	1.67 (0.98–2.84)	0.061
Hypertension	0.98 (0.62–1.55)	0.94
Age group 7: 80 and older		
No hypertension	1.27 (0.70–2.32)	0.44
Hypertension	0.75 (0.45–1.26)	0.28

^a^ Adjusted for cancer, age, sex, dyslipidemia, hypertension, diabetes, heart disease, education, household income, and significant effect modifiers (cancer age and cancer hypertension).

## 4 DISCUSSION

In this analysis of a cross‐sectional survey of adult Canadians, we examined the association between prior/active cancer and prevalence of self‐reported stroke. We found that age and hypertension significantly modified the association between cancer and self‐reported stroke, and the association tended to be stronger in younger adults and those without hypertension. The association between cancer and the prevalence of self‐reported stroke was most pronounced for adults under the age of 40 years. Our findings suggest that cancer may be an important risk factor for stroke, particularly among younger individuals without hypertension. Our results support a growing body of evidence pointing toward a stronger association between cancer and stroke among younger individuals.^16^

Our findings may in part be explained due to differences in the distribution of tumor types and treatment‐associated sequelae across age groups. Prevalent cancer types among young adults, such as Hodgkin lymphoma, have well‐established associations with long‐term vascular complications, including stroke, coronary artery disease, cardiomyopathy and valvular heart disease.^17^, ^18^ This vascular toxicity is in part secondary to specific therapeutic modalities such as mantle radiation.^19^, ^20^ Cancer‐associated coagulopathy due to both the underlying disease and chemotherapy might also play a more prominent role in the development of stroke among young patients who otherwise lack conventional cardiovascular risk factors.

Malignancy exerts its prothrombotic effects through various mechanisms, including venous stasis, release of prothrombotic substances by tumor cells,^21^, ^22^ induction of inflammatory responses, and inhibition of fibrinolysis.^23^, ^24^ It follows that the underlying risk factors leading to stroke in patients with cancer might be different from those in patients without cancer.^25^ In the present study, we found that the association between cancer and self‐reported stroke was greatest among a subpopulation that is traditionally thought to have the lowest risk for stroke (i.e., the youngest age strata without hypertension). The prevalence stroke increases with age due to several factors, including vascular risk factors which can function as confounders for the association between cancer and stroke. In our study, the relationship between cancer and stroke was masked until an interaction between age and cancer were considered. Furthermore, since the relationship between cancer and stroke is not uniform across all ages, effect modification of age must be considered. While previous studies have reported that stroke patients with active cancer tend to be younger than those without cancer,^26^ to our knowledge, no studies have looked at age as an effect modifier of this complex relationship.

The strengths of our study include our large sample size, use of a representative national sample, careful control of confounding and detection of interaction using multiple logistic regression, and the use of weighted proportions/measures of association to reflect the complex sampling technique. The result is a sample that is representative of 98% of the entire Canadian population. The major limitation to our study is the cross‐sectional nature of our data. Given that the CCHS survey did not collect data about the timing of stroke relative to cancer diagnosis, it is possible that self‐reported stroke occurred prior to the diagnosis of cancer precluding conclusions about a causal relationship between stroke and cancer. Other limitations to our study include the potential for self‐reporting bias: age‐related misclassification of chronic conditions may be higher in younger individuals, which can bias away from the null.^27^ Moreover, self‐reporting bias could have occurred due to increased contact with the healthcare system among respondents with a current or prior diagnosis of cancer, including participants receiving cancer treatments who may be more familiar with their medical history and more likely to report a prior diagnosis of stroke. Third, the CCHS survey data stand alone and are not linked to registry data or database diagnoses. Unfortunately, this means that neither cancer nor stroke diagnoses can be confirmed and are based on self‐reported patient diagnoses. Self‐reported data may also be subject to recall bias and survival bias, thus our sample may represent milder/more benign forms of both diseases. There may also be underreporting of stroke due to symptomless survival, and information collected with respect to some covariates may lack specificity (e.g., “heart disease”). Furthermore, the CCHS does not collect details surrounding the type/severity of stroke or treatment‐related factors (i.e., receipt of thrombolysis/thrombectomy), all of which are important prognostic factors for survival. Lastly, although CCHS collects information about current or past cancer diagnoses, it does not distinguish between different cancer types or stages. As a result, we were unable to explore whether our findings varied according to cancer type across age groups, or whether the effect modification by age was attributable to a particular cancer type. Given the heterogeneous spectrum of cancer and its variable association with different stroke subtypes,^25^, ^28^ these factors should be further evaluated in future studies.

## 5 CONCLUSION

Active or prior cancer is associated with an increased prevalence of self‐reported stroke in the Canadian population, particularly among younger adults who lack conventional vascular risk factors like hypertension.

## AUTHOR CONTRIBUTIONS

**Ronda Lun:** Conceptualization (lead); investigation (lead); methodology (lead); software (lead); writing – original draft (lead); writing – review and editing (lead). **Joseph R. Shaw:** Conceptualization (lead); formal analysis (equal); investigation (equal); methodology (equal); validation (equal); writing – original draft (equal); writing – review and editing (lead). **Danielle Roy:** Methodology (equal); software (equal); writing – review and editing (equal). **Deborah Siegal:** Writing – review and editing (equal). **Tim Ramsay:** Writing – review and editing (equal). **Yue Chen:** Conceptualization (equal); data curation (equal); methodology (equal); resources (equal); software (equal); supervision (equal); writing – review and editing (equal). **Dar Dowlatshahi:** Conceptualization (equal); supervision (equal); writing – review and editing (equal).

## FUNDING INFORMATION

None.

## CONFLICT OF INTEREST STATEMENT

None.

## ETHICS STATEMENT

None required.

## CONSENT STATEMENT

None.

## Supporting information


**Data S1:** Table S1.

## Data Availability

This study used cross‐sectional data from the 2015‐2016 iteration of the Canadian Community Health Survey (CCHS), conducted by Statistics Canada. This data is publicly accessible.
