# Supplementary material for: Effect modification of age and hypertension on cancer and prevalence of self‐reported stroke – A cross‐sectional study
Source: Cancer Med. 2023 Apr 21;12(11):12518–23. doi: 10.1002/cam4.5964 (PMC10278503; doi:10.1002/cam4.5964)
Supplement: Supplementary file 1 — Data S1: Table S1. [file CAM4-12-12518-s001.pdf]

Supplemental Materials

eTable 1. Prevalence of self-reported stroke according to covariates in Canadian adults from 2015-2016.

N=86809	**Number of cases[%]**	**Sample size for each category[%]**	**%[&]**	**P-value[&]**
**Cancer**				
• Yes	230	7100	2.88%	<0.0001
• No	1057	79709	1.00%	
**Age**				
• 18-29	19	11816	0.33%	<0.001
• 30-39	25	13298	0.18%	
• 40-49	67	12523	0.63%	
• 50-59	160	15887	0.72%	
• 60-69	333	16956	1.83%	
• 70-79	371	10782	3.48%	
• ≥80	312	5547	5.79%	
**Sex**				
• Male	640	39574	1.24%	0.0079
• Female	647	47235	0.99%	
**Smoking status**				
• Non-smoker	475	43812	0.91%	<0.0001
• Former smoker	577	27043	1.62%	
• Current smoker	235	15954	1.00%	
**Dyslipidemia**				
• Yes	521	13780	3.21%	<0.0001
• No	766	73029	0.80%	
**Hypertension**				
• Yes	785	19783	3.66%	<0.0001
• No	502	67026	0.56%	
**Diabetes**				
• Yes	380	7618	4.40%	<0.0001
• No	907	79191	0.86%	
**Heart Disease**				
• Yes	470	5641	7.85%	<0.0001
• No	817	81168	0.79%	
**Education level**				
• No post-secondary education	737	33095	1.70%	<0.0001
• Completed post-secondary education	550	53714	0.80%	
**Racial background**				
• White	1208	76833	1.22%	<0.0001

• Non-white	79	9976	0.75%	
**Annual household income**				
• < $60,000	919	38746	1.81%	<0.0001
• ≥ $60,000	368	48063	0.72%	

**Statistically significant association (p<0.05)*

% *Unweighted results were used for frequencies*

& *Weighted results were used for proportions and p-statistic to adjust for complex survey design*
